# Editorial: Burden of Illness in People With Epilepsy: From Population-Based Studies to Precision Medicine

**DOI:** 10.3389/fneur.2018.01164

**Published:** 2019-01-09

**Authors:** Adam Strzelczyk, Karl Martin Klein, Felix von Podewils

**Affiliations:** ^1^Epilepsy Center Frankfurt Rhine-Main and Department of Neurology, Goethe-University Frankfurt, Frankfurt am Main, Germany; ^2^Epilepsy Center Hessen and Department of Neurology, Philipps-University Marburg, Marburg, Germany; ^3^LOEWE Center for Personalized Translational Epilepsy Research (CePTER), Goethe-University Frankfurt, Frankfurt am Main, Germany; ^4^Department of Clinical Neurosciences, University of Calgary, Calgary, AB, Canada; ^5^Epilepsy Center Greifswald and Department of Neurology, Ernst-Moritz-Arndt-University Greifswald, Greifswald, Germany

**Keywords:** epilepsy, seizure, anticonvulsant, antiepileptic drug (AED), quality of life, imaging

Epilepsy is a common and chronic neurological disease that is characterized by recurrent seizures which impose a major burden on patients, their caregivers, and society (1, 2). The aim of this Research Topic was thus to provide evidence that personalized translational epilepsy research will benefit patients through targeted experimental (3), clinical and network research (4, 5). There is a fast growing number of publications that deal with personalized or precision approaches for the treatment of epilepsy, Figure 1.

**Figure 1:**
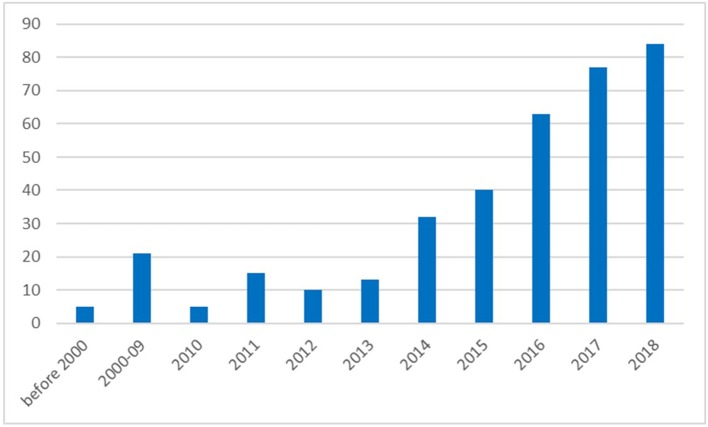
Number of publications listed in PubMed referring to search terms “Epilepsy” and “personalized” or “precision medicine” (PubMed query on September 13th 2018).

People with epilepsy face disease-specific restrictions concerning self-sufficiency, mobility, career choice, family planning, and other social aspects (6, 7). An analysis of patients with severe drug-refractory epilepsy showed a seven-fold increase in mortality, along with high costs and frequent epilepsy-related accidents and injuries (Strzelczyk et al.). The authors used a German health insurance database to administer a top-down approach, drawing patients from a representative cohort and matched them to a cohort that was not affected. Comorbidities, like depression and vascular disorders, were significantly increased in patients with epilepsy (Strzelczyk et al.). Focusing on epilepsy-related accidents and injuries, Willems et al. used a bottom-up approach in a cross-sectional study, and it showed that there was a possibility of a reduced quality of life and increased depression scores in affected patients (Willems et al.). The presented data in both studies fits well with other recent burden-of-disease studies that showed the costs for hospital treatment and anticonvulsants as being major cost drivers (8–11) and that persisting seizures were also associated with a reduced quality of life (12, 13).

While the use of newer anticonvulsants may be associated with increased costs, it also has the potential to significantly reduce the seizure burden. Brivaracetam is the latest anticonvulsant that has been approved as an add-on therapy for the treatment of focal-onset seizures (14). A single-center study from Marburg in Germany (Zahnert et al.) shows promising post-marketing results in a cohort of mainly drug-refractory patients, while another multi-center study focused on patients with epileptic encephalopathies (Willems et al.). In both studies, 50% responder rates of 35 to 45% were achieved, which were well in line with other postmarketing results (15–17).

The initial response to anticonvulsants is explored in patients with newly diagnosed epilepsy, and showed that the initial 6-month response, as well as the number of seizures prior to treatment and brain-imaging abnormalities, are important prognostic factors (Xia et al.).

Lifestyle-dependent factors are important, especially in genetic generalized epilepsies. Until now, it was unclear as to whether alcohol consumption has an impact on epilepsy in these patients (18). Even if the risk is generally increased in patients with epilepsy, patients with genetic generalized epilepsies have a particularly high risk of alcohol related-seizures after the consumption of a large amount of alcohol. This can also be attributed to accompanying factors, such as altered sleep architecture or impaired adherence to antiepileptic medication (Hamerle et al.).

The genetic architecture of common non-lesional focal epilepsies was evaluated in a study that uses a customized panel of 21 well-known focal epilepsy genes (Tsai et al.). The study revealed that only 1.85% (11/593 patients) carried pathogenic or likely pathogenic variants in these genes, and this indicated that other yet to be discovered genes play a role as well.

Comorbidities, such as cognitive issues, are often present in patients with epilepsy (19). Gorny et al. showed that not all scales that were used to assess global cognitive function, work reliably in patients with epilepsy.

Structural abnormalities associated with clinical and neuropsychological characteristics in genetic epilepsies was evaluated in two studies on juvenile myoclonic epilepsy (JME) using MRI diffusion tensor imaging (DTI), and in benign childhood epilepsy, with centrotemporal spikes (BECTS), which used the graph theory analysis based on the cortical gyrification index, respectively (Domin et al.; Jiang et al.). In JME, the extent of microstructural abnormalities within the subcortical networks, including cortico-cortical, thalamo-frontal, and cortico-spinal connections, determined the clinical manifestation and subtype of JME in the individual patient, such as photoparoxysmal responses or seizures with predominant motor symptoms (Domin et al.). In contrast, abnormal cortical folding that was mainly in the central region is presumably the neuroanatomical basis for BECTS (Jiang et al.). The findings of both studies are important steps for the establishing of the pathophysiological concepts found in genetic epilepsies.

A step forward in the care for people with epilepsy could be through the introduction of technological therapies. Page et al. pointed out in their perspective article that there has been an increase in patient-triggered interventions, a finding based on automated monitoring of indicators and risk factors facilitated by technological advances. The main goal of such interventions would be the reduction of epilepsy-related mortality with SUDEP (Sudden Unexpected Death in Epilepsy) being the main reason for epilepsy-related deaths (20, 21).

An important point on this road is the development of an automatic computer-based detection algorithm of seizures. In their review, Baumgartner et al. described the use of potential bio signals, such as scalp EEG, ECG, and surface EMG, which can be combined for an algorithm and implemented into devices. The daily work of clinicians may be significantly improved by the use of an automated long-term EEG review. This was described by Koren et al. as an automatic critical care EEG pattern detection method that would be helpful in reducing review times.

This Research Topic presents a compilation of different studies which increases the visibility of the high burden associated with epilepsy, along with providing some directions as to how personalized or precision approaches may help to overcome this burden. In the coming years we will see a dramatic increase in personalized or precision medicine, Figure 1, that will significantly contribute to the management of epilepsy.

## Author Contributions

All authors listed have made a substantial, direct and intellectual contribution to the work, and approved it for publication.

### Conflict of Interest Statement

The authors declare that the research was conducted in the absence of any commercial or financial relationships that could be construed as a potential conflict of interest.
